# Comparative Liquid Biopsy Testing for *KRAS* Mutations From Plasma Cell‐Free DNA (cfDNA) and Extracellular Vesicles in Lung Adenocarcinoma

**DOI:** 10.1002/cnr2.70517

**Published:** 2026-03-11

**Authors:** Caeli J. Zahra, Tian Mun Chee, Edward K. H. Stephens, Elizabeth J. Keir, Brielle A. Parris, Hannah E. O'Farrell, Anita F. Goldsworthy, Rayleen V. Bowman, Ian A. Yang, Kwun M. Fong

**Affiliations:** ^1^ UQ Thoracic Research Centre, Faculty of Health, Medicine and Behavioural Sciences The University of Queensland Brisbane Australia; ^2^ Thoracic Medicine, The Prince Charles Hospital, Metro North Hospital and Health Service Brisbane Australia

**Keywords:** ddPCR, extracellular vesicles, *KRAS*, liquid biopsy, lung adenocarcinoma, plasma

## Abstract

**Background:**

Liquid biopsy has emerged as a promising, minimally invasive tool to detect cancer biomarkers. Extracellular vesicles (EVs) are secreted into biofluids to transport cargo such as DNA and are a potential biosource for liquid biopsy biomarkers. To determine the optimal use of liquid biopsy for diagnosing mutations in lung adenocarcinoma (LUAD), we compared the *KRAS* mutation status in DNA isolated from four different plasma fractions, including EVs.

**Methods:**

Plasma was collected from 58 participants diagnosed with LUAD (early‐stage (I, II), *n* = 30; late‐stage (IIIB, IV), *n* = 28) with known *KRAS* mutation (*KRAS*mt*)* or wild‐type *KRAS* (*KRAS*wt). Three distinct plasma‐derived fractions were prepared by sequential differential ultracentrifugation aiming to isolate EVs (pellets 1, 2, 3 (P1–P3)). These were tested together with the corresponding plasma supernatant (SUP) for the presence of *KRAS G12/G13* mutations by droplet digital PCR (ddPCR).

**Results:**

In early‐stage *KRAS*mt LUAD, mutations were detected by ddPCR in only 1 of 15 processed plasma supernatant samples and 1 of 15 P3 samples, but not in any P1 or P2 pellets. In late‐stage *KRAS*mt LUAD, mutations were detected in 13/14 of SUP samples, but only in a small fraction of the pellet preparations: P1 (0/14), P2 (2/14) and P3 (2/13) samples.

**Conclusion:**

We conclude that ddPCR testing of plasma supernatant achieved high overall agreement with tumour *KRAS*mt status. There was varying abundance of *KRAS* detected in the plasma fractions.

**Abbreviations**
cfDNA: cell‐free DNA
ctDNA: circulating tumour DNA
ddPCR: droplet digital PCR
EGFR: epidermal growth factor receptor
*KRAS*
: *Kirsten rat sarcoma* gene
*KRAS*mt: *Kirsten rat sarcoma* G12/G13 mutation positive tumour
*KRAS*wt: *Kirsten rat sarcoma* G12/G13 wildtype tumour
LUAD: lung adenocarcinoma
MFA: mutation fractional abundance
P1–3: pellet 1–3
PBS: phosphate‐buffered saline
SUP: plasma supernatant
TEM: transmission electron microscopy
TRPS: tunable resistive pulse sensing

## 1 Introduction

Lung cancer is the leading cause of cancer‐related mortality globally [1], and lung adenocarcinoma (LUAD) is the most common subtype, with a representation of 50% amongst all non‐small cell lung cancers [2]. In clinical practice, detecting oncogenic driver mutations in LUAD is crucial to guide treatment decisions by oncologists. Mutation testing in liquid biopsies emerges as an attractive, minimally invasive technique to detect oncogenic driver mutations [1, 3]. To date, two FDA‐approved in vitro diagnostic tests, namely COBAS *EGFR* Mutation Test v2 (Roche) and Idylla TM ctEGFR Mutation Assay, are used as companion testing for *EGFR* mutations in plasma samples [4]. These tests show good inter‐laboratory reproducibility with varying sensitivity ranging from 86.7% to 100% and specificity from 95.7% to 100% in plasma samples [5, 6]. In some instances, the low abundance of tumour DNA in peripheral blood limited the mutation test sensitivity in plasma samples [3, 7].

Extracellular vesicles (EVs) are naturally released nanoparticles that play essential roles in intercellular communication [8, 9]. EVs are found in all biofluids (plasma, urine, pleural fluid, etc.) and are reported to transport molecular cargo such as DNA to recipient cells [9, 10, 11]. EVs have been conventionally classified as exosomes, microvesicles and apoptotic bodies based on biogenesis and size [8, 9, 10, 12, 13, 14]. Exosomes (30–150 nm) are secreted by fusion of multivesicular bodies with the plasma membrane of the cell [9, 14]. Microvesicles (100–1000 nm) are formed from the outward budding of the cellular membrane and are secreted after fission from the plasma membrane [9, 14]. Apoptotic bodies (50–5000 nm) are produced from the outward budding of the plasma membrane of a cell undergoing programmed cell death (apoptosis) [14, 15]. Despite well‐defined classification of EVs based on biogenesis, isolating a pure EV population is challenging even with specialised techniques such as ultracentrifugation or column‐based exclusion chromatography. In recognition of this, recent MISEV2023 guidelines [16] encourage the use of broader nomenclature, such as large EVs (> 200 nm) and small EVs (< 200 nm), to describe a crude EV sample isolated by ultracentrifugation, column‐based chromatography or ultrafiltration methods.

Tumour cells produce and release substantial amounts of EVs into the bloodstream that drive pathogenesis and establish pre‐metastatic niches in distant tissues and organs [10, 11]. Tumour‐derived circulating EVs may be enriched with oncogenic biomarkers that are useful for diagnostic applications [8, 11].

Previous studies in colon cancer and pancreatic cancer have shown higher test sensitivity for *KRAS (Kirsten rat sarcoma)* mutation detection in EV DNA than in cell‐free DNA (cfDNA) (66.7%–85% versus 45.5%–70%, respectively) [17, 18]. Furthermore, a previous study determined that exosome nucleic acids (RNA and DNA) combined with cfDNA have a higher sensitivity for detecting EGFR mutations in non‐small cell lung cancer plasma than cfDNA alone [19]. A recent study showed that in advanced‐stage lung cancer, small EV DNA was not preferentially enriched in tumour DNA for shallow whole genome sequencing [20]. Notwithstanding the difficulty of isolating a particular pure EV population, sequential differential centrifugation may produce enrichment of EV populations according to buoyant density. We therefore hypothesised that different plasma fractions enriched for different sized EVs vary in DNA abundance, including tumour DNA, and that in LUAD, oncogenic driver mutations may be more readily detectable in DNA isolated from certain plasma EV fractions than others when using highly sensitive techniques [9, 11]. To explore this, we applied droplet digital PCR (ddPCR) to determine DNA abundance and *KRAS* mutation status in DNA derived from four different plasma fractions obtained by sequential differential centrifugation commonly used to isolate EVs.

## 2 Material and Methods

### 2.1 Participants and Ethics Approval

This study was approved by Human Research and Ethics committees at The University of Queensland (2 020 000 442/50265) and TPCH (HREC/2020/QPCH/50265). Plasma samples were obtained with written informed consent from 58 donors diagnosed with LUAD and were archived at The Prince Charles Hospital (TPCH) UQTRC Lung Bank (HREC/17/QPCH/54). Tumour *KRAS* mutation status was obtained from routine molecular ancillary testing (next‐generation sequencing) on diagnostic small lung tumour biopsies and fluids or resected lung tumour tissue (Supporting Information: Supplementary Methods).

### 2.2 Plasma Fractionation Processes

**FIGURE 1:**
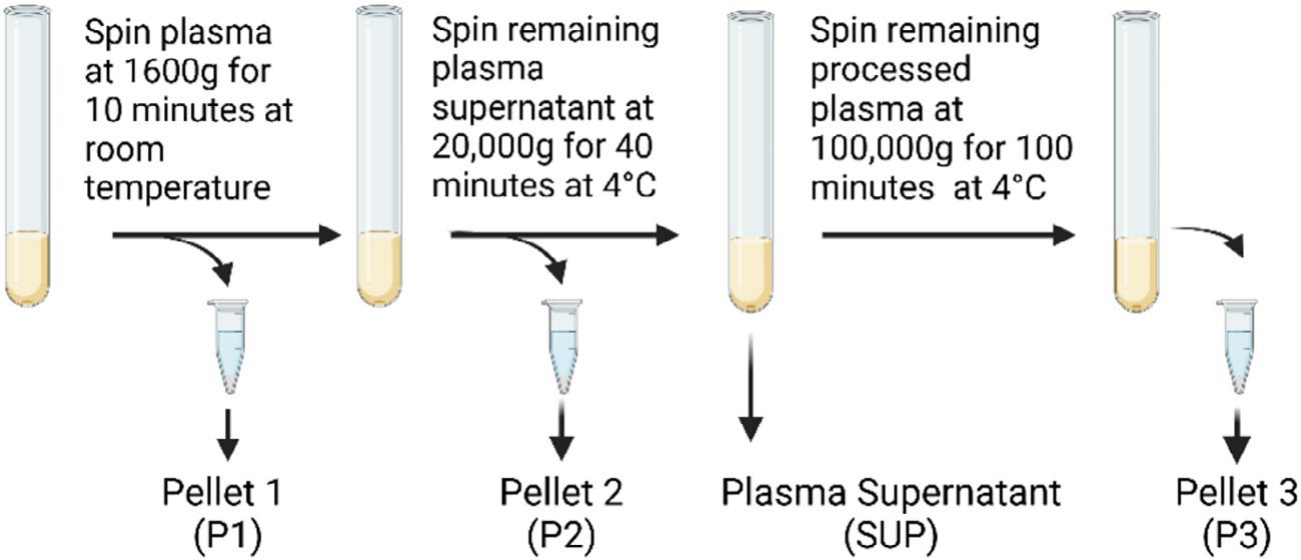
Differential centrifugation to isolate P1, P2, SUP and P3 from plasma. Following the separation of plasma from erythrocytes and buffy coat, the plasma was spun at 1600 x g for 10 min to isolate pellet 1 (P1), followed by 20 000 x g for 40 min to isolate pellet 2 (P2). The resulting supernatant was designated as plasma supernatant (SUP). P1, P2 and SUP were stored at −80°C for downstream experimentation and completed prior to this study. Upon retrieval, a final centrifugation was performed on plasma supernatant at 100 000× g for 1 h and 40 min at 4°C to isolate pellet 3 (P3).

Peripheral whole blood was spun at 1200 × g for 10 min at room temperature to separate plasma from buffy coat and erythrocytes. Plasma (5–10 mL) was spun at 1600 × g for 10 min at room temperature using an Eppendorf centrifuge 5810R (Hamburg, Germany), generating *Pellet 1 (P1)*, which was resuspended in 100 uL of retained plasma. The resulting supernatant underwent a second centrifugation at 20 000 × *g* for 40 min at 4°C using an Eppendorf centrifuge 5810R (Hamburg, Germany), generating *Pellet 2 (P2)*, which was resuspended in 100 μL of plasma. The resulting *plasma supernatant (SUP)*, as well as pellets P1 and P2, were stored at −80°C for downstream experiments. Upon retrieval from storage, 2.5 mL of the SUP fraction was spun at 100 000 × g for 1 h and 40 min (accumulated centrifugal effect, ω^2^t = 5.46e10) at 4°C using a Beckman Optima XPN‐100 ultracentrifuge, and a 50.2 Ti rotor (Beckman Coulter, CA, USA) to generate *Pellet 3 (P3)*. Pellet 3, and the thawed Pellet 1 and 2, were resuspended in 1 mL 1X Phosphate‐Buffered Saline (1X PBS) and underwent DNA extraction immediately. The plasma fractionation processes are illustrated in Figure 1.

### 2.3 Western Blotting of EV Markers

Protein quantification and western blotting were performed as previously described [21]. Briefly, 2 μg of protein from plasma fractions P1, P2, SUP, P3 of a randomly selected representative sample were stained against Anti‐Albumin (1:1000, Cell Signalling Technology, Cat. 4929S), Anti‐FLOT1 (D2V7J) XP Rabbit mAb (1:1000; Cell Signalling Technology, Cat. 18634S) and Anti‐CD9 (D8O1A) Rabbit mAb (1:2000, Abcam, Cat. EPR2949). Flotillin‐1 and CD9 were developed using SuperSignal West Femto Maximum Sensitivity Substrate (ThermoFisher Scientific, Cat. 34 094) and Albumin was developed using SuperSignal West Pico PLUS chemiluminescent Substrate (ThermoFisher Scientific, Cat 34 579) for 5 min, and imaged as previously described [21].

### 2.4 Tunable Resistive Pulse Sensing (TRPS)

The particle size distribution and concentration of plasma pellets (P1, P2, P3) were measured by tunable resistive pulse sensing (TRPS) using IZON Science's Exoid as per the manufacturer's specifications (V1.0.0.181). NP100 and NP600 nanopores were used with measurable target size ranges of 50–330 nm and 275–1570 nm, respectively. These nanopores were operated with a stretch of 47 mm and 400 mV current, with the samples analysed at three different pressures of 200, 400 and 800 Pa. The assay was calibrated to experimental parameters using CPC100 and CPC800 calibration particles for NP100 and NP600 nanopores, respectively. The size range and percentage population were graphed using IZON Data Suite software V1.0.2.32.

### 2.5 Transmission Electron Microscopy (TEM)

EV visualisation by TEM was performed at UQ Centre for Microscopy and Microanalysis (UQCMM). Negative staining was performed on undiluted P1‐P3 samples fixed with 4% EM‐grade formaldehyde. The images were acquired using a JEOL JEM 1400 Flash transmission electron microscope with an OEM 2 k × 2 k CMOS camera at 80 to 120 kV with drift correction active and scale bars of 200 nm.

### 2.6 DNA Extraction

DNA was extracted from plasma fractions (SUP, P1, P2, P3) using Plasma/Serum cfc‐DNA/cfc‐RNA Advanced Fractionation Kit (NORGEN BIOTEK CORP, Cat. 68 300) as per the manufacturer's specifications. In the final step, DNA was eluted in 50 μL of Elution Buffer F. A further clean‐up step was performed using The Monarch PCR and DNA Cleanup Kit (New England BioLabs Cat. T1030S) following the manufacturer's specifications. Briefly, DNA was diluted at a ratio of 1:5 with DNA Cleanup Binding Buffer, transferred to a column, and spun at 16 000 *x g* for 1 min at room temperature. The samples were washed twice with 200 μL of DNA Wash Buffer before elution in 8 μL of DNA Elution Buffer. Two microlitres of the eluted DNA was quantified using Qubit 4 Fluorometer (ThermoFisher Scientific, Cat. Q33226) with Qubit High Sensitivity DNA (ThermoFisher Scientific, Cat. No. Q32851).

### 2.7 ddPCR Experiments

All plasma fractions (P1, P2, SUP, P3) were tested with the ddPCR *KRAS G12/G13* screening kit (Bio‐Rad, CA, USA, Cat. 1 863 506) and analysed using the QX100 ddPCR system (Bio‐Rad) with a manual droplet generator following the manufacturer's specifications. Briefly, plasma fractions, non‐template and positive template controls were emulsified with droplet generator oil (Bio‐Rad, CA, USA Cat. 186 300) to generate up to 20 000 droplets. The droplets were then transferred to a ddPCR 96‐well plate (Bio‐Rad, CA, USA Cat.12001925) for thermal cycling using c1000 touch thermal cycler (Bio‐Rad) at recommended settings: Step 1°C–95°C for 10 min, Step 2–40 cycles of 94°C for 30 s, 55°C for 1 min and Step 3°C–98°C for 10 min. The plate was left at 4°C for 30 min prior to detection using a QX100 droplet reader (BioRad, CA, USA). Data was analysed using QX Manager software V1.1, applying appropriate thresholding for each tested sample. Based on a Bio‐Rad protocol [22], a false positive rate assay was also performed, from which it was determined that the minimum criterion to classify a sample as *KRAS* mutant was the observation of ≥ 3 *KRAS* mutation‐positive events (see Figure S1).

### 2.8 Statistical Analysis

Agreement analysis between *KRAS* mutation status reported in pathology molecular testing results and the *KRAS* mutation testing results by ddPCR in each plasma fraction was performed using Fisher's exact test in GraphPad Prism (V.10.2.2), with a 95% confidence interval and a *p* value of < 0.05. Concordance between plasma and tumours was analysed using Cohen's Kappa (*k*) with GraphPad Prism online, with strong agreement indicated by a k‐value > 0.8, compared with a null of 0.60. Mutation fractional abundance (MFA) was obtained from QX Manager software (Version 1.1), which calculates the percentage of *KRAS* mutant‐positive events from the total number of *KRAS*‐positive events (wildtype and mutant). Heat maps demonstrating MFA with positive events stratified by mutant or wildtype *KRAS* were prepared using GraphPad Prism (V.10.2.20). The number of *KRAS* DNA copies in each sample was analysed using one‐way ANOVA with Tukey multiple comparisons, with a 95% confidence interval and a *p* value of < 0.05.

## 3 Results

### 3.1 Study Cohort

Table 1 shows the demographics of the 58 participants. Forty‐one (70.7%) were female, and 17 (29.3%) were male, with a median age of 68.1 years. Fifty‐four participants were treatment naïve prior to their surgery, whilst 4 participants had chemotherapy or radiation therapy for previous unrelated cancers of the lung (*n* = 2) or non‐lung origin (*n* = 2) at a minimum of 10 months prior. Six participants (10.3%) had never smoked, while 52 participants (89.7%) were either former or current smokers at the time of surgery, with a median cumulative pack‐years of 37. Twenty‐nine (50%) LUAD cases carried tumour *KRAS G12/G13* mutations (*KRAS*mt), whilst 29 were wildtype for *KRAS G12/G13* (*KRAS*wt). In each arm of *KRAS*mt or *KRAS*wt, 15 cases were early‐stage LUAD (I or II) and 14 were late‐stage LUAD (IIIB or IV).

**TABLE 1:** Participant demographics of study cohort (*n* = 58) for *KRAS* mutation testing in plasma fractions with ddPCR.

Participants characteristics	Cohort	
	*n*	%
Sex		
Female	41	70.7
Male	17	29.3
Age at diagnosis, years		
Median (range)	68.1 (48.0–85.7)	
*KRAS* mutation status and TNM staging		
*KRAS* mutation positive	29	50
– Early tumour stage (I, II)	15	51.7
– Late tumour stage (IIIB, IV)	14	48.3
*KRAS* wild‐Type (no mutation)	29	50
– Early tumour stage (I, II)	15	51.7
– Late tumour stage (IIIB, IV)	14	48.3
Smoking status		
Smoker, former or current	52	89.7
Never	6	10.3
Pack years^a^		
Median (range)	37 (5–100)	

Abbreviation: TNM, tumour, node, metastasis.

^a^ Past and current smokers.

### 3.2 Evidence of the Presence of Extracellular Vesicles in Plasma Fractions

**FIGURE 2:**
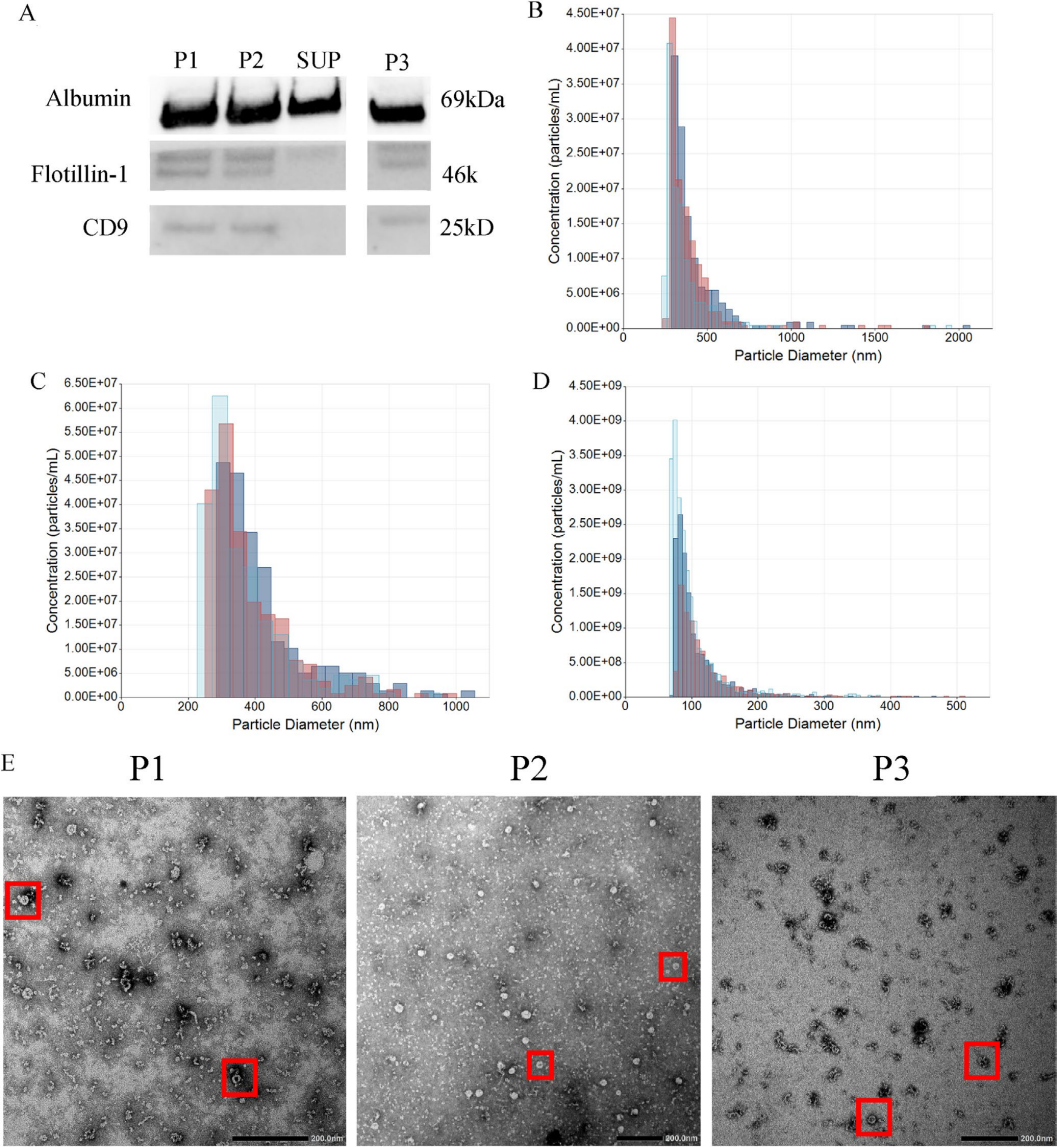
EV characterisation through western blot for protein contents, TRPS for size distribution of particles and TEM for morphology and size confirmation. (A) Western blot of negative control marker, albumin and EV markers Flotillin‐1 and CD9 in a representative cancer patient. P1–3 = pellet 1–3, SUP = plasma supernatant. (B) The pellets were measured using 400 mV of current, variable pressures and a suitable nanopore at a 47 mm stretch after calibration with known standards. P1 sample was measured with a NP600 nanopore at 200 Pa (light blue), 400 Pa (red) and 800 Pa (dark blue). (C) P2 sample was measured with a NP600 nanopore at 200 Pa (light blue), 400 Pa (red) and 800 Pa (dark blue). (D) P3 was measured with a NP100 nanopore at 200 Pa (light blue), 400 Pa (red) and 800 Pa (dark blue). (E) TEM shows isolated EVs in each fraction with varying sizes as indicated by the red boxes and scale bars of 200 nm.

In a representative sample, EV characterisation was performed on all plasma pellets (P1, P2, P3) and the plasma SUP fraction. In western blot (Figure 2A), Flotillin‐1 expression was observed in P1, P2 and P3 fractions, with a very faint band present in the SUP fraction. P1, P2 and P3 fractions all showed low CD9 expression, whereas no CD9 expression was observed in the plasma SUP fraction. High expression of Albumin was observed in all plasma fractions (P1, P2, SUP, P3). These results suggest that EVs were present in P1, P2, SUP and P3 fractions, albeit with a diluted presence of EVs in the SUP fraction.

Analyses by TRPS showed that the particle size distribution ranged from 255 to 2057 nm (Figure 2B–D), and cup‐shaped morphology in P1, P2 and P3 fractions was demonstrated by TEM (Figure 2E). The mean particle sizes (mean ± SD) observed for P1 at 200 , 400 , 600 Pa was 367 ± 177.2 nm, 394 ± 195.3 nm and 424 ± 208.4 nm, respectively, with a collective range of 255–2057 nm. P2 fraction had a mean particles size (mean ± SD) of 370 ± 125.4 nm at 200 Pa, 388 ± 124.1 nm at 400 Pa and 429 ± 142.9 nm at 600 Pa with a range of 255–2051 nm. The particle size (mean ± SD) for the P3 fraction was 99 ± 42.1 nm, 107 ± 41.6 nm and 117 ± 48.8 nm at 200 Pa, 400 Pa and 600 Pa, respectively, with a range of 69–511 nm. The raw concentrations reported for P1, P2 and P3 fractions were 1.26E+08, 2.18E+08 and 1.4 E+10 particles/ml, respectively. TEM confirmed the presence of EVs in all plasma pellet fractions (Figure 2E). Taken together, observations from western blotting analysis, TRPS and TEM provide evidence of the presence of EVs in plasma pellets—P1, P2 and P3.

### 3.3 All Plasma Fractions Contained *KRAS* *G12/* *G13* DNA

**FIGURE 3:**
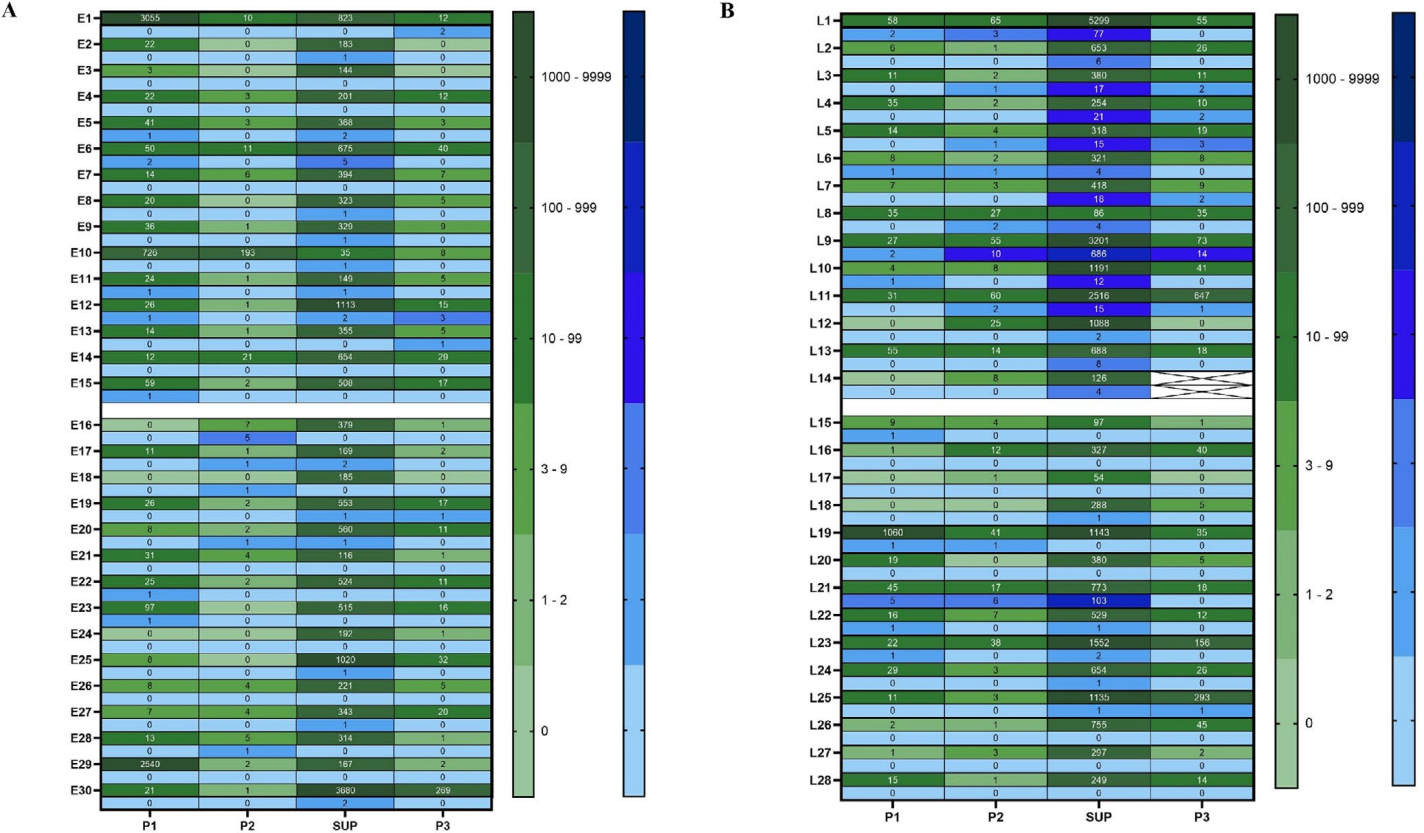
Heat maps denoting ddPCR droplet counts for the number of *KRAS* wildtype (green top row) or mutation‐positive events (blue bottom row) for each case plotted against P1, P2, SUP and P3 in columns, for (A) early‐stage LUAD cases (*n* = 30) and (B) late‐stage LUAD cases (*n* = 28). The number of positive events detected by ddPCR was labelled in the relevant cell. No detected events are in the lightest shades of blue or green; the second lightest shaded indicate inconclusive result (1–2 events). The remaining shades of blue and green are detected events, with the darkest shade having the highest number of detected events. Early‐stage LUAD samples E1‐15 are pathologically confirmed *KRAS*mt, with E16‐30 being *KRAS*wt. Late‐stage LUAD samples L1‐L14 are *KRAS*mt and L15‐28 are *KRAS*wt.

The DNA extracted from 58 cases and quantified by Qubit HS DNA assay showed that DNA detectability varied between different plasma fractions, with the greatest amounts detected in the plasma SUP fraction of late‐stage LUAD. The number of cases with measurable DNA for SUP, P1, P2 and P3 fractions was 28, 8, 7 and 4 (out of 30 early‐stage LUAD), whereas amongst 28 cases with late‐stage LUAD, the numbers with detectable DNA were 28, 10, 10 and 10, respectively (see Table S1). However, *KRAS G12/G13* DNA copies were detectable even in samples with unquantifiable DNA content by Qubit HS DNA assay. *KRAS G12/G13* wildtype DNA was observed in SUP, P1, P2 and P3 fractions in ddPCR experiments. As shown in Figure 3, the number of cases with at least one wildtype or mutant event detection in SUP, P1, P2 and P3 fractions was 30, 27, 24 and 27 for early‐stage LUAD (*n* = 30), and 28, 24, 26 and 25 for late‐stage LUAD (*n* = 28), respectively.

**FIGURE 4:**
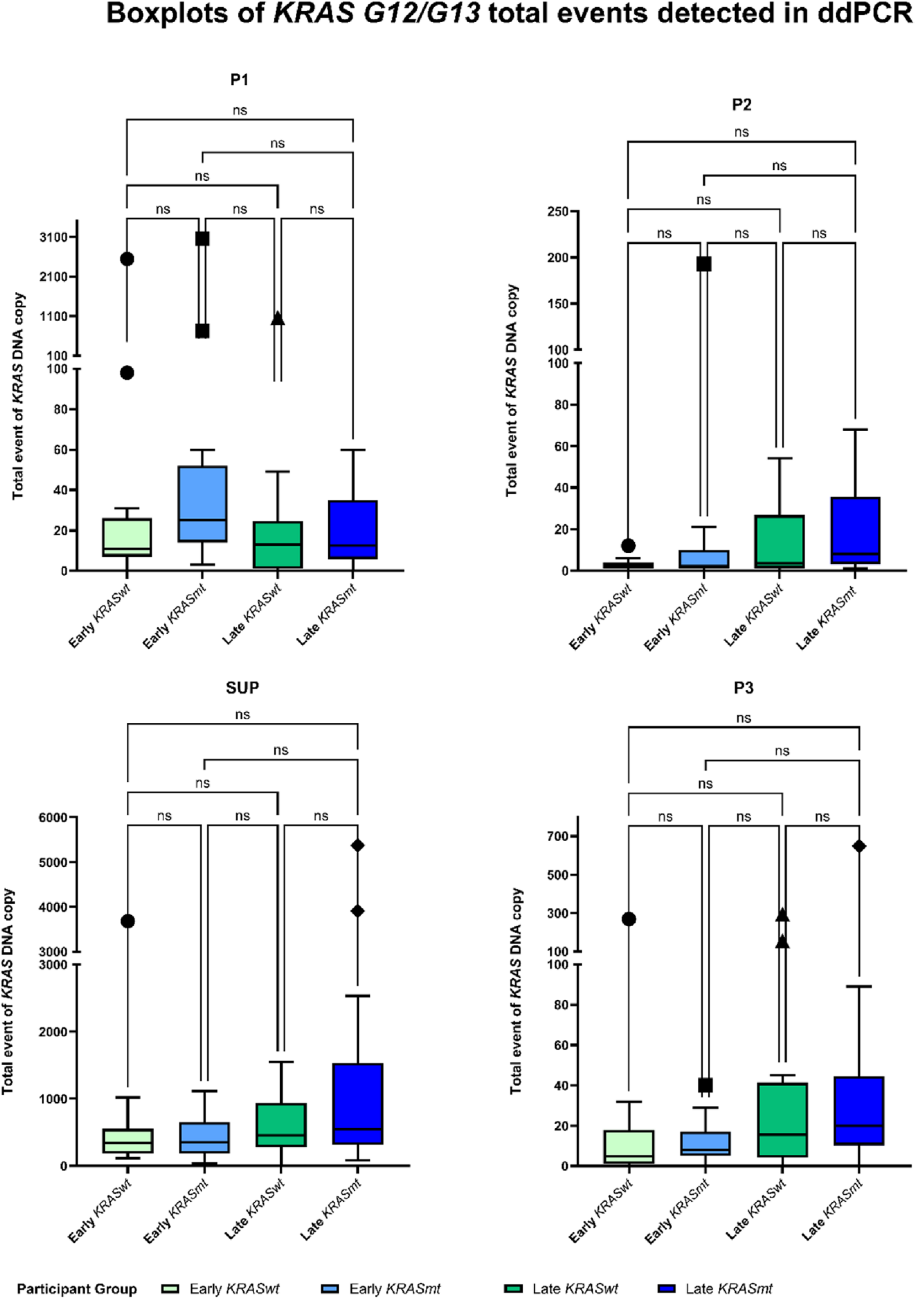
Total number of *KRAS G12/G13* DNA copies detected in P1, P2, SUP and P3 in 58 LUAD cases. Light green circles indicate early‐stage wildtype *KRAS* LUAD, while the light blue squares indicate early‐stage mutant *KRAS* LUAD. Dark green and triangles indicate late‐stage wildtype *KRAS* LUAD, while the dark blue diamonds indicate late‐stage mutant *KRAS* LUAD. SUP = plasma supernatant, P1 = pellet 1, P2 = pellet 2, P3 = pellet 3, LUAD = lung adenocarcinoma.

Figure 4 shows boxplots of total events (wildtype and mutant) of *KRAS G12/G13* DNA copies detected in 58 LUAD cases, stratified by tumour stage and *KRAS G12/G13* mutation status. In all SUP, P2 and P3 fractions, a consistently higher number of *KRAS G12/G13* DNA copies (wildtype or mutant) were detected in late‐stage LUAD than in early‐stage LUAD, although the difference was not statistically significant. In P1 fraction, the *KRAS*wt arm shows similar levels of total events between the early *KRAS*wt and late *KRAS*wt arms, whereas a higher number of total events was observed in the early *KRAS*mt arm than in the late *KRAS*mt arm. Comparing amongst the plasma fractions, SUP fractions had a much higher number of total events than P1, P2 or P3 fractions. The number of total events in P1, P2 and P3 fractions of the 58 LUAD cases was mostly in the range of 0–100, and a relatively small number of cases had > 100 detected total events (see Table S2). These results show that SUP fractions were most abundant in *KRAS G12/G13* DNA copies.

### 3.4 All Plasma Fractions Showed Potential for *KRAS* *G12/G13* Mutation Testing

The 58 LUAD cases were stratified as early‐stage *KRAS*mt (E1‐E15), early‐stage *KRAS*wt (E16‐E30), late‐stage *KRAS*mt (L1‐L14) and late‐stage *KRAS*wt (L15‐L28). Applying detection thresholds of ≥ 3 positive events (See Supporting Information: Supplementary Results) to *KRAS G12/G13* mutation testing by ddPCR, the plasma SUP fraction showed greater test sensitivity than P1, P2 and P3 fractions. Mutation detectability in *KRAS*mt LUAD was higher in late‐stage than early‐stage cases as shown in Table 2. In *KRAS* tumour mutant cases, concordance between tumour pathology results and plasma ddPCR testing was observed in two early‐stage cases—E6 (SUP), E12 (P3) and in all except one late‐stage case (L12). Specifically, 13 out of 14 late‐stage cases had detectable *KRAS G12/G13* mutations in SUP fractions (L1‐L11, L13‐L14), as well as in P2 (L1, L9) and P3 (L5 and L9). Of note, the P3 fraction of L14 was excluded from the mutation analyses due to technical errors.

**TABLE 2:** The number of samples for each plasma fraction that showed at least 3 *KRAS* mutation‐positive events with ddPCR for early‐stage and late‐stage participants.

	Early‐Stage LUAD participants		Late‐stage LUAD participants	
	Wildtype *KRAS*	Mutant *KRAS*	Wildtype *KRAS*	Mutant *KRAS*
P1	0/15	0/15	1/14	0/14
P2	1/15	0/15	1/14	2/14
SUP	0/15	1/15	1/14	13/14
P3	0/15	1/15	0/14	2/13

**FIGURE 5:**
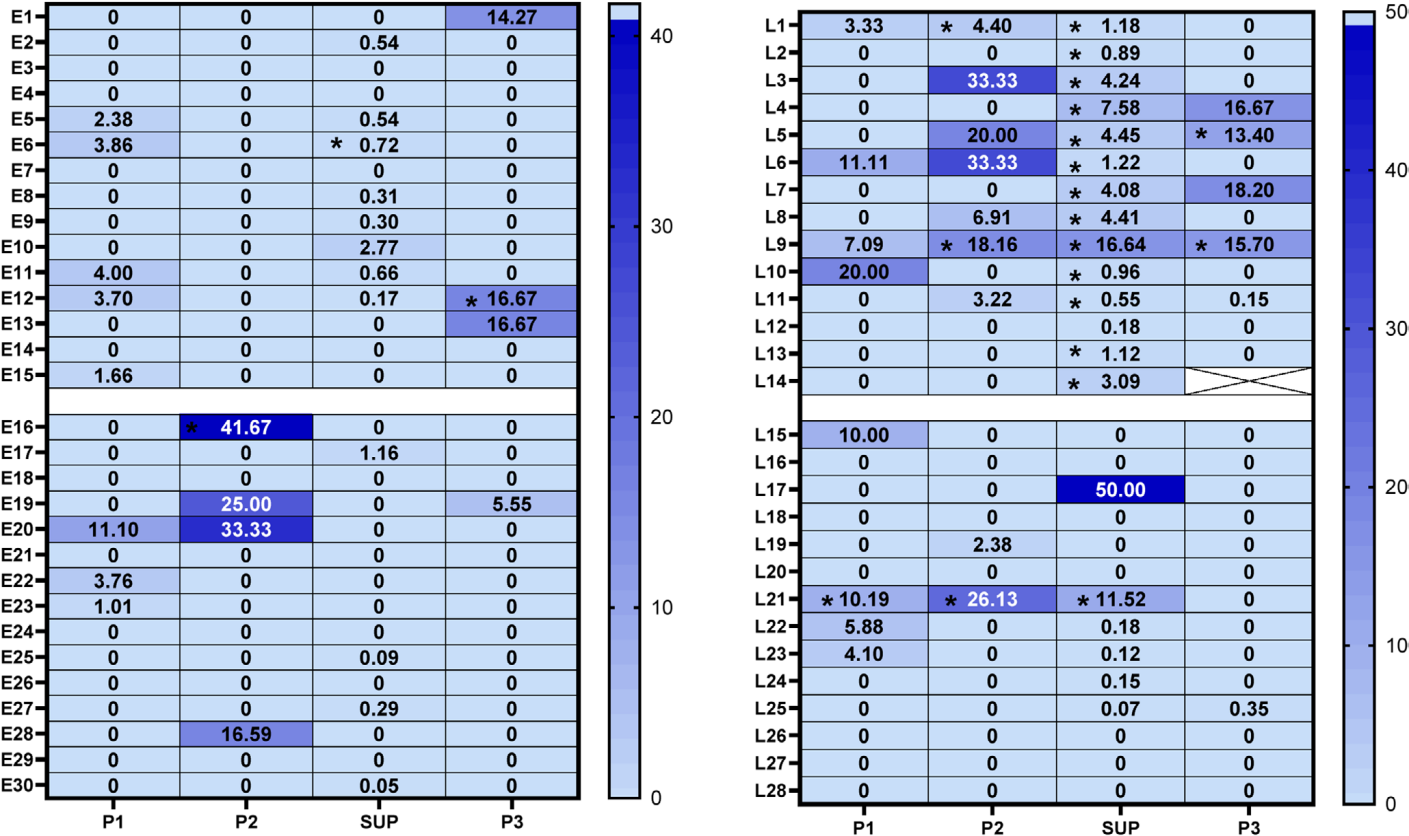
Heat map denoting ddPCR Mutation Fractional Abundance (MFA) for each case plotted against P1, P2, supernatant plasma and P3 in columns, for (A) early‐stage LUAD participants (*n* = 30) and (B) late‐stage LUAD participants (*n* = 28). The shades of blue denote the mutation fractional abundance, with the darkest shade having the highest MFA while the lightest shade has no MFA calculated. Early‐stage LUAD samples E1‐15 are pathologically confirmed *KRAS*mt participants, with E16‐30 being *KRAS*wt participants. Late‐stage LUAD samples L1‐L14 are *KRAS*mt cases and L15‐28 are *KRAS*wt cases; * indicate samples that were considered positive by containing > 3 *KRAS* mutant drops.

Nonetheless, there were also some false positive results with 2 out of 29 cases whose tumours were *KRAS* wildtype as determined by pathology testing showing mutations in various plasma fractions, for example SUP, P1 and P2 in the late‐stage LUAD case L21. Next, ddPCR results were interrogated further by MFA as seen in Figure 5. All plasma fractions (SUP, P1, P2, P3) in all 58 participants showed some level of MFA, but only a subset met the detection threshold of ≥ 3 *KRAS*mt events (Figure 3). Mutation detectability (defined as the number of *KRAS* mutant positive LUAD cases returning positive events above the threshold) was greatest in SUP fractions. However, P1, P2 and P3 fractions showed higher MFA (range: 4.4–41.67, mean: 18.29, SD: 11.34) than the corresponding SUP fractions (range: 0.55–16.64, mean: 4.18, SD: 4.59), suggesting that P1, P2, P3 fractions contain *KRAS G12/G13* mutant DNA at a higher abundance compared to background wildtype *KRAS* DNA than SUP fractions.

Agreement analyses describing sensitivity, specificity, positive percentage agreement (PPA), negative percentage agreement (NPA), overall percentage agreement (OPA) and Cohen's kappa (*κ*) for P1, P2, SUP and P3 fractions against tumour mutation status are presented in Table 3. Overall, plasma mutation testing by ddPCR showed greater sensitivity in late‐stage LUAD cases, but comparable specificity between early and late‐stage LUAD. Respectively, the sensitivities and specificities observed for late‐stage LUAD in SUP, P2 and P3 fractions ranged from 14.3% to 92.9% and 92.9% to 100%, whereas for early‐stage LUAD the ranges were 0%–6.7% and 93.8%–100%. For P1 fractions, both early and late‐stage LUAD showed 0% sensitivity but a specificity of 100% (early‐stage) and 92.9% (late‐stage). In early‐stage LUAD, the concordance in *KRAS* mutation detection between ddPCR results and pathologically confirmed tumour status in all plasma fractions did not achieve statistical significance (*p* > 0.99, *ĸ* = < 0.07), whereas in late‐stage LUAD, strong overall concordance was observed in SUP fractions (*p* < 0.0001, *ĸ* = 0.857), but not in P1, P2 or P3 fractions (*p* > 0.99 *ĸ* = 0, *p* > 0.99 *ĸ* = 0.071 and *p* = 0.22 *ĸ* = 0.159, respectively).

**TABLE 3:** Agreement and concordance analyses for the detection of *KRAS* mutation status by ddPCR in plasma fractions versus tumour mutation status. The samples were considered in agreement with tumour mutation status of *KRAS* mutant when 3 or more *KRAS* mutant events were detected by ddPCR analysis. Statistical analysis was completed using Fisher's Exact test and Cohen's kappa.

		Early‐stage (I/II)	Late‐Stage (IIIB/IV)
P1	Sensitivity (%)	0	0
	Specificity (%)	100	92.9
	PPA (%)	0	0
	NPA (%)	50	48.2
	OPA (%)	50	46.4
	*ĸ*	0	0
	*p*	> 0.99	> 0.99
P2	Sensitivity (%)	0	14.3
	Specificity (%)	93.8	92.9
	PPA (%)	0	66.7
	NPA (%)	48.3	52.0
	OPA (%)	46.7	53.6
	*ĸ*	0.07	0.07
	*p*	> 0.99	> 0.99
SUP	Sensitivity (%)	6.7	92.9
	Specificity (%)	100	92.9
	PPA (%)	100	92.9
	NPA (%)	51.7	92.9
	OPA (%)	53.3	92.9
	*ĸ*	0.07	0.86
	*p*	> 0.99	< 0.0001
P3	Sensitivity (%)	6.7	15.4
	Specificity (%)	100	100
	PPA (%)	100	100
	NPA (%)	51.7	56.0
	OPA (%)	53.3	53.3
	*ĸ*	0.07	0.16
	*p*	> 0.99	0.22

Abbreviations: *ĸ* = kappa coefficient, NPV = negative positive agreement, OPA = overall percentage agreement, PPA = positive percentage agreement.

## 4 Discussion

Mutation detection in plasma presents an attractive, minimally invasive liquid biopsy. Current FDA‐approved methods primarily employ real‐time PCR and next‐generation sequencing to detect lung cancer driver mutations [23]. Furthermore, biomarker discovery utilising EVs has emerged as another bioresource for mutation detection due to unique EV biogenesis, strong influence over tumour development and progression, as well as over the tumour microenvironment under pathological conditions [24]. In this study, EV P1‐3 were successfully characterised using western blotting, TEM and TRPS to confirm EV morphology, size and concentration. Despite the smallest particle measured with TRPS being 255 nm in these fractions, TEM analysis showed many small EVs present in P1 and P2, likely due to the lower limit of the nanopore selected for TRPS analysis being around 255 nm. The nanopore NP600, was selected to accommodate the analysis of larger EVs because a smaller nanopore would have been blocked by particles, resulting in incomplete analysis of the samples.

Mutations in the *KRAS* gene are the most prevalent in LUAD amongst western populations [25, 26]. The Agilent Resolution ctDx FIRST assay was FDA‐approved in 2022 for testing the *KRAS G12C* mutation, as a companion diagnostic for *KRAS G12C* small molecule inhibitor therapies [27], and clinical trials are underway using in vitro diagnostic tests for *KRAS* mutations [28]. Recent studies have demonstrated potential for EV‐based mutation detection in various body fluids, including plasma [17, 18, 29]. ddPCR is a sensitive technique for the detection of mutations in liquid biopsy, particularly plasma [30], and can analyse genetic biomarkers from different sources including cfDNA and nucleic acids derived from EVs [17, 29, 30]. Here, we tested various plasma fractions, including EV‐enriched fractions, to explore the varying abundance of *KRAS* DNA in these fractions using ddPCR. Overall, SUP plasma from late‐stage LUAD had higher levels of cfDNA compared to early‐stage LUAD as indicated by the total number of events detected. *KRAS* mutation detection rates were poor in all plasma fractions from early‐stage cases and in all pellet fractions from late‐stage LUAD. However, in late‐stage LUAD, *KRAS* mutations were highly detectable (92.9%) in the SUP fraction. Our results align with a previous study in which 89% of late‐stage patients had detectable genomic variants in cfDNA from plasma [25]. One major challenge in testing plasma samples for tumour‐related mutations in early‐stage disease where tumours may be resectable or curable is that the amount of DNA in plasma is often insufficient, as reported in pancreatic cancer [31]. In small number of late‐stage lung cancer cases studied by whole genome sequencing small EV DNA was found to be 12 times less abundant (per mL of plasma) than cfDNA [20]. In comparison, in pancreatic cancer (localised, locally advanced and metastatic cases) higher mutation detection rates were found in small EV‐derived DNA (66.7%, 80% and 85%, respectively) than in cfDNA (45.5%. 30.8% and 57.9%) [18]. Despite the higher mutation detection rate of exosome‐derived DNA in pancreatic cancer [18], we did not find P3 (putatively containing small EVs) to be the superior plasma fraction to test for *KRAS* mutation in LUAD. This is potentially due to the different EV isolation methods utilised in the pancreatic cancer study, which included treating EV pellets with DNase, followed by a PBS wash and a second isolation with ultracentrifugation, producing a more concentrated and purer EV pellet. It is worth noting that the success of an EV‐based protein test in detecting early‐stage cancer has been found to vary according to cancer type—pancreatic cancer having the highest at 95.5% success, followed by 74.4% in ovarian cancer and 43.8% in bladder cancer [32], so this inherent variability between cancers could be a fundamental consideration in determining the suitability of EV‐based tests for early‐stage cancer. In the future, a larger starting plasma volume together with refining methods to further improve sample purity and to select tumour‐specific EVs are all potentially worthy steps towards optimisation of minimally invasive methods of tumour mutation detection in lung and other cancers.

To further explore our hypothesis, sensitivity, specificity and concordance were calculated. Our findings show that the overall specificity of ddPCR *KRAS* mutation testing on plasma fractions for early and late‐stage LUAD was 92.9%–100%. Further, we showed in late‐stage LUAD that the sensitivity in the SUP fraction was significantly higher than in EV‐enriched fractions. In our investigation of *KRAS* mutation detection by ddPCR in early‐stage LUAD, the sensitivity was similar between processed plasma and P3, whereas a study in colon cancer patients across a range of disease stages [17] demonstrated higher sensitivity in EV DNA (76.7%) than in cfDNA (70%). We found concordance in *KRAS* mutation status between ddPCR on plasma fractions and next‐generation sequencing (routine molecular ancillary testing) of the tumour in only a limited number of early‐stage *KRAS*mt LUAD cases (3%). Moreover, only late‐stage LUAD had strong concordance results for the SUP fraction, with all other fractions having slight agreement. A previous study published comparable results, reporting that *KRAS* genotyping with ddPCR in late‐stage LUAD tumours had a concordance (*k*) of 0.72 with cfDNA [33]. In comparison, a study of early‐stage resected pancreatic cancers reported 95.5% and 68.2% concordance for plasma EV DNA and ctDNA, whereas concordance for fine needle tumour aspirates was 83.3% and 66.8% for plasma EV DNA and ctDNA, respectively [34]. The lack of concordance in either early or late‐stage LUAD for the plasma fraction putatively containing the largest EVs (P1) suggests that this fraction is unsuitable for KRAS mutation testing. The overall low sensitivity and discordant results seen in the early‐stage cases across all plasma fractions are likely due to the inherent biological and technical limitations of isolating low amounts of cfDNA.

Mutation Fractional Abundance (MFA) measures the presence of mutant DNA alleles amongst wildtype background DNA and can be useful in cancer diagnosis and monitoring of disease. We found a higher MFA in EV fractions than in corresponding SUP fractions, despite SUP having the greater DNA concentration. Similar results were reported in a study comparing *BRAF* V600E mutation test performance by ddPCR between circulating tumour DNA (ctDNA) and small EV‐associated DNA [35] which found higher total DNA copies but comparable mutation fractional abundance between plasma ctDNA and small EV‐associated DNA [35]. Another study found that EVs had a lower concentration of DNA than cell‐free plasma but higher MFA at 0%–83.8% and 0%–45.3%, respectively [17]. Based on the findings from different studies and ours, it can be postulated that while EV‐enriched plasma fractions can be more abundant in mutant DNA copies, the overall low DNA content in the EV‐enriched fractions hinders ddPCR test sensitivity. Until more effective EV isolation methods are available, unfractionated plasma might be a feasible and more viable bioresource for biomarker studies. Our study shows that MFA is a promising way to detect mutations amongst wild‐type EV DNA. Together with refined methods of isolating a larger and purer yield of EVs, MFA could be a useful way of providing insight into disease status and progression.

False positive results were observed in some cases, especially in L21 where three out of four tested fractions showed the presence of *KRAS G12/G13* mutation. In this case, insufficient primary tumour material was available for mutation analysis, but the pathology of pleural fluid with 5% tumour content was consistent with metastatic adenocarcinoma, and no mutation was detected by next‐generation sequencing in the extracted DNA. Inconsistencies with the ddPCR results could be due to technical artifacts, biological factors, sampling bias or tumour heterogeneity [3, 36]. Any non‐specific amplification, potentially due to biological variations, may have resulted in increased fluorescence in the droplets mimicking the appearance of mutant alleles to the ddPCR system. This is normally demonstrated as ‘rain’ in the sample [22], in the case of L21, only the SUP fraction showed minimal rain with P1‐P3 containing no rain. Contamination is another potential source causing false positive; however, each ddPCR assay showed no contamination in the non‐template controls. Furthermore, clonal haematopoiesis‐derived mutations could also cause false positive *KRAS* mutations to be detected with ddPCR [37]. As it is possible that tumour subclone representation differs between pleural fluid and EVs or plasma, the ddPCR detected *KRAS* mutation in the plasma fractions could have originated from KRAS mutant clonal tumour elements that were not detected in pleural fluid. Similarly, tumour heterogeneity and sampling bias can cause discordant results where the section of tumour being tested for mutations does not have oncogenic driver mutations while other subclone cells within the tumour have oncogenic driver mutations present. This will show a negative result for the oncogenic driver in the first test, but upon retesting a different section of tumour, the mutation can be present [3, 36]. It has previously been demonstrated that plasma analysis with ddPCR can unveil mutations not detected in the tumour samples [30, 38]. It is worth noting that without completing multiregional sequencing on the primary tumour, tumour heterogeneity, sampling bias and clonal haematopoiesis cannot be confirmed as the reason for the discordant results. There was no sample remaining for re‐analysis with ddPCR or to investigate further with highly sensitive techniques like next‐generation sequencing.

One of the limitations of this study was that the volume of plasma (2.5 mL) used to generate the various fractions was relatively small when compared to the amount of plasma attainable from venepuncture for prospective mutation testing. Also, comparison between neat plasma and the various derived plasma fractions could have provided more accurate information about the test sensitivity of *KRAS G12/G13* mutation status using ddPCR. Another consideration is that although blood samples were collected, processed and stored according to protocols applying to blood and tissue biobanking, these procedures were not specifically optimised for this particular study. The MISEV suggests that by preprocessing plasma to remove large EVs, cells and co‐isolated particles, as in deriving P1 and P2, cell‐free plasma would remain and therefore a purer P3 (small EVs) could then be isolated [16]. This limitation resulted in EVs in P1/P2 being isolated prior to storage and P3 being isolated after sample thawing. As P1/P2 were resuspended in remaining plasma and not stored in a different buffer, the pellets were all frozen in plasma and thawed once; thus, storage duration and conditions did not differ between the fractions significantly. Further to this limitation, resuspending pellets in remnant plasma resulted in co‐isolation of cfDNA and albumin contamination in P1 and P2 samples limiting the interpretation of EV isolation methods and their ability to generate pure EV DNA samples. Although the presence of EVs in P1‐P3 fractions was confirmed in a representative case, comprehensive quantitation of the proportions of EV DNA and free circulating DNA in plasma was not determined in every case. Additionally, in order to prioritise overall EV yield, the isolated population of EVs was examined as a whole, rather than selecting for tumour‐specific EVs. Nevertheless, with further optimisation of sample volume input, fractionation procedures and the challenge of EV subpopulation selection, examination of a tumour‐specific EV subpopulation could be additionally informative in future studies. Another consideration is that isolating EVs using ultracentrifugation has limited clinical applications due to the complexity of the technique, which would not be suitable for general clinical use, especially as a more advanced method is required to increase the sensitivity of oncogenic driver mutation detection. Further to this notion, size exclusion chromatography methods often isolate EVs with higher purity than ultracentrifugation methods. We have previously compared EV isolation methods (ultracentrifugation and size exclusion chromatography) for pleural fluids, observing a variable amount of EV recovery and purity [21]. Both methods were considered prior to this experiment (data not published), and we found similar characteristics based on western blotting, nanoparticle analysis and DNA yield. More efficient yet effective EV isolation methods that are suitable for clinical settings while maintaining a high sensitivity and specificity would be highly desirable. Although many studies have demonstrated the potential of EV nucleic acids (DNA, RNA, miRNA) to differentiate disease states [39], to distinguish between tumour and non‐tumour [40, 41], and to predict disease aggressiveness, progression and responses to treatment [31, 42, 43], our focus here was on the detection of *KRAS G12/G13* DNA content only with a relatively simple method. Finally, it is noteworthy that digital PCR technology has evolved rapidly over the past decade, and more advanced digital PCR systems for mutation testing may provide improved performance in due course.

## 5 Conclusions

In summary, we have explored *KRAS G12/G13* mutation testing using ddPCR in various plasma fractions, some of which were enriched for EVs, in LUAD. We have demonstrated varying abundance of cfDNA in these fractions and corroborated the utility of plasma genotyping with ddPCR in late‐stage LUAD. The biological constraint of low cfDNA in early‐stage LUAD was seen in this study; however, there was evidence of *KRAS* DNA seen in each plasma fraction. The observations from this study will help inform the development of more refined methods and hypotheses for future studies.

## Author Contributions

**Caeli J. Zahra:** conceptualisation, methodology, validation, formal analysis, investigation, resources, data curation, writing – original draft, writing – editing and reviewing, visualisation; **Tain Mun Chee:** conceptualisation, methodology, validation, formal analysis, resources, writing – editing and reviewing, visualisation, Supervision; **Edward K. H. Stephens:** formal analysis, writing – editing and reviewing; **Elizabeth J. Keir:** formal analysis, writing – editing and reviewing; **Brielle A. Parris:** methodology, writing – editing and reviewing; **Hannah E. O'Farrell:** methodology, writing – editing and reviewing; **Anita F. Goldsworthy:** data curation, Writing – editing and reviewing; **Rayleen V. Bowman:** methodology, formal analysis, writing – editing and reviewing, visualisation; **Ian A. Yang:** conceptualisation, methodology, validation, formal analysis, resources, writing – editing and reviewing, visualisation, supervision; **Kwun M. Fong:** conceptualisation, methodology, validation, formal analysis, resources, writing – editing and reviewing, visualisation, supervision, project administration, funding acquisition.

## Funding

This work was supported by the National Health and Medical Research Council, 1164020.

## Conflicts of Interest

The authors declare no conflicts of interest.

## Supporting information


**Data S1:** cnr270517‐sup‐0001‐supinfo.docx.

## Data Availability

The data that support the findings of this study are available from the corresponding author upon reasonable request.
