# Supplementary material for: Comparative Liquid Biopsy Testing for KRAS Mutations From Plasma Cell‐Free DNA (cfDNA) and Extracellular Vesicles in Lung Adenocarcinoma
Source: Cancer Rep (Hoboken). 2026 Mar 11;9(3):e70517. doi: 10.1002/cnr2.70517 (PMC12977295; doi:10.1002/cnr2.70517)
Supplement: Supplementary file 1 — Data S1: cnr270517‐sup‐0001‐supinfo.docx. [file CNR2-9-e70517-s001.docx]

**Online Supplementary Appendix**

**Comparative liquid biopsy testing for *KRAS* mutations from plasma cell-free DNA (cfDNA) and extracellular vesicles in lung adenocarcinoma.**

**AUTHORS:**

Caeli J. Zahra^a^, Tian Mun Chee^a^, Edward K. H. Stephens^a^, Elizabeth J. Keir^a^, Brielle A. Parris^a^, Hannah E. O’Farrell^a^, Anita F. Goldsworthy^a,b^, Rayleen V. Bowman^a,b^, Ian A. Yang^a,b^ and Kwun M. Fong^a,b^.

**AFFILIATIONS**

^a^UQ Thoracic Research Centre, Faculty of Health, Medicine and Behavioural Sciences, The University of Queensland, Brisbane, Australia

^b^Thoracic Medicine, The Prince Charles Hospital, Metro North Hospital and Health Service, Brisbane, Australia

**Supplementary appendix content:**

Supplementary Methods: pages 2-5

Supplementary Results: page 6

Supplementary Figure legend 1: page 6

Supplementary Figure 1: page 7

**SUPPLEMENTARY METHODS**

**Patient blood collection and processing:** Peripheral blood was collected from 58 participants and fractionated into supernatant (SUP), P1, P2 and P3 plasma fractions as below. Briefly, blood was collected into 2-5 BD vacutainer K2 EDTA plus blood collection tubes. The whole blood was spun at 1200 x *g* for 10 minutes at room temperature to execute blood fractionation. The plasma is transferred into a separate 10mL tube for further processing of pellets or frozen for further downstream experiments. The buffy coat and erythrocytes were combined and transferred to 5mL tubes for storage at -80°C.

**P1 isolation:** Peripheral blood was spun to separate plasma from the buffy coat and erythrocytes. P1 were isolated from this unprocessed plasma using centrifugal force. Briefly, the plasma was spun at 1600 x g for 10 minutes at room temperature to isolate P1. The supernatant (plasma) was removed without disturbing the pellet and processed further, the P1 sample was resuspended in 100µL of the remaining plasma. The P1 sample was stored separately at -80°C for further downstream experiments. Upon retrieval, the pellet was resuspended in 1mL of PBS.

**P2 isolation:**  P2 samples were isolated from the remaining plasma following P1-depletion by centrifugation at 20,000 x g for 40 minutes at 4°C. The resulting supernatant (plasma supernatant) and P2 pellet (resuspended in approximately 100 μL of plasma) were stored at -80°C for downstream applications. Upon retrieval, the pellet was resuspended in 1mL of PBS.

**Pathologically determined mutation status of patients:** The mutation status of the patients was confirmed by Queensland Pathology using sequencing techniques on resected tumour samples. At least 20ng of patient samples underwent DNA extraction using the QIAamp DNA investigator kit (QIAGEN, Hilden, Germany). DNA extracted from samples before August 2020 underwent next generation sequencing with the TruSight Tumour panel. This panel detects several mutations including *EGFR* (exons 18, 19, 20, 21), *ERBB2* exon 20, *KRAS* (exons 2, 3, 4, 6, *NRAS* (exons 2, 3, 4, 5) and BRAF exons 11 and 15. This assay has a 97.3% analytical sensitivity with a variant threshold detection at 3% and minimum 1000-fold depth. The currently used (September 2020 onwards) next-generation sequencing is on a miSeq using an AmpliSeq Illumina custom DNA panel. The custom panel can detect mutations in, *KRAS*, *EGFR*, *MET*, *ERBB2* and *BRAF*. Specifically, mutations in the following gene exons: *KRAS* exons 2, 3, 4 and 6, *EGFR* exons 1, 3, 5, 7, 9, 11, 13, 15, 18, 19, 20, 21, 23, 25, and 27, MET exons 2, 5, 6, 9, 11, 14, 16, 17, 18, 19 and 21, *ERBB2* exons 1, 3, 4, 6, 9, 10, 12, 13, 16, 18, 19, 20, 21, 23 and 26, and *BRAF* exons 11 and 15. This sequencing assay has an analytical sensitivity of 98.8% and a variant threshold of 5% with a minimum read depth of 500. The results of the mutation testing are reported to the referring practitioner.

**Statistical analysis:** A Fisher’s exact test with sensitivity, specificity and predictive values was employed to compare early-stage *KRAS* mutation positive tumours and late-stage *KRAS* mutation positive tumours with their respective stage controls. Overall percentage agreement was calculated by the number of total agreed/concordant readings divided by the total number of all readings. Kappa coefficient was calculated using GraphPad prism ‘Quantity agreement with Kappa’ online service. All analyses used a 95% confidence interval with statistical significance defined as p<0.05 and graphed using GraphPad Prism V9.0 (GraphPad, CA, USA).

| **New method** | **Reference method*** | | |
| --- | --- | --- | --- |
|  | **Test positive** | **Test negative** | **Row total** |
| **Test positive** | A (TP) | B (FP) | A + B |
| **Test negative** | C (FN) | D (TN) | C +D |
| **Column total** | A +C | B + D | A + B + C +D |
| **Sensitivity** | TP/(TP + FN) = A/(A + C) | | |
| **Specificity** | TN/(FP + TN) = D/(B + D) | | |
| **PPA** | TP/(TP + FP) = A/(A + B) | | |
| **NPA** | TN/(TN + FN) = D/(C + D) | | |
| **OPA** | 100% x (A + D)/(A + B + C + D) | | |

**SUPPLEMENTARY RESULTS**

**False positive rate of the ddPCR G12/G13 *KRAS* Screening Kit:** The *KRAS* *G12/G13* screening kit has a standard protocol that has been optimised by Bio-Rad^1^, however, the false positive rate (FPR) still needed to be determined for individual experiments. The FPR of the *KRAS G12/G13* screening kit was important to estimate prior to ddPCR analysis to determine the threshold for calling a sample mutation-positive or negative. The FPR was estimated by the average number of ‘mutant’ positive events detected in 4 wells of wild-type only sample (loaded at 5ng). As seen in **Supplementary Figure 1**, the FPR for the *KRAS G12/G13* screening kit at 5ng of tissue DNA was two events per well.

**SUPPLEMENTARY FIGURE LEGENDS**

Supplementary Figure 1: The determined false positive rate (FPR) of the *KRAS* screening kit using ddPCR: The number of mutation-positive events in wild-type (WT) only sample (n = 4) was averaged to estimate the FPR of the *KRAS G12/G13* Screening Kit. Data presented as mean±SD of the four replicates present on the left of the dotted line as individual values.

Supplementary Table 1: QC of DNA using Qubit 4 Fluorometer before mutation status analysis with ddPCR. Samples were quantified after concentration and immediately before analysis with ddPCR. For samples quantified on 1/9/2022 and 2/9/2022, Qubit assay standard 1 was 88.71 RFU while standard 2 was 38256.83 RFU. For samples quantified on 5/9/2022, Qubit assay standard 1 was 139.83 RFU while standard 2 was 38079.93 RFU. For samples quantified on 17/03/2023, Qubit assay standard 1 was 81.65 RFU while standard 2 was 42810.48 RFU. For samples quantified on 20/03/2023, Qubit assay standard 1 was 87.96 RFU while standard 2 was 44234.16 RFU. For samples quantified on 23/03/2023, Qubit assay standard 1 was 95.33 RFU while standard 2 was 44787.96 RFU. For samples quantified on 13/04/2023, Qubit assay standard 1 was 93.08 RFU while standard 2 was 44726.65 RFU. SUP = plasma supernatant, P1-P3 = Pellet 1-3 L1-14 = Late stage KRAS mutant lung adenocarcinoma participants, L15-28 = Late stage wildtype KRAS lung adenocarcinoma participants, E1-15= Early stage KRAS mutant lung adenocarcinoma participants, E16-30 = Early stage KRAS wildtype lung adenocarcinoma participants, Too Low = means that the 1x dsDNA high sensitivity Qubit assay could not determine the DNA concentration for the sample as it was outside the range set by the standards (<0.02ng/µL).

Supplementary Table 2: The descriptive statistics (central tendency and spread) of total events (WT+MT) for early and late stage stratified into wild type and mutant KRAS tumours. MT = *KRAS* mutant, WT = *KRAS* Wildtype, LUAD = lung adenocarcinoma. SUP = plasma supernatant, P1-3 = pellet 1 – Pellet 3

**Supplementary Figure 1:**

**Supplementary Table 1:**

| Date | Sample | DNA concentration (ng/µL) | | | |
| --- | --- | --- | --- | --- | --- |
|  |  | SUP | P1 | P2 | P3 |
| 1/09/2022 | L1 | 9.35 | 0.07 | 0.07 | 0.11 |
| 1/09/2022 | E16 | 0.32 | 0.04 | Too Low | Too Low |
| 1/09/2022 | E1 | 1.40 | 3.84 | Too Low | Too Low |
| 1/09/2022 | E17 | 0.17 | Too Low | Too Low | Too Low |
| 1/09/2022 | E2 | 0.51 | Too Low | Too Low | Too Low |
| 1/09/2022 | E3 | 0.34 | Too Low | Too Low | Too Low |
| 1/09/2022 | E18 | 0.42 | Too Low | 0.19 | 0.07 |
| 2/09/2022 | E4 | 0.34 | Too Low | Too Low | Too Low |
| 2/09/2022 | E5 | 0.24 | Too Low | 0.13 | Too Low |
| 2/09/2022 | E6 | 0.54 | Too Low | Too Low | Too Low |
| 2/09/2022 | E19 | 0.38 | Too Low | Too Low | Too Low |
| 2/09/2022 | E7 | 0.32 | Too Low | Too Low | Too Low |
| 2/09/2022 | E8 | 0.23 | Too Low | 0.10 | Too Low |
| 2/09/2022 | E9 | 0.28 | Too Low | Too Low | Too Low |
| 2/09/2022 | E10 | Too Low | 0.51 | 0.13 | Too Low |
| 2/09/2022 | E20 | 0.51 | Too Low | 0.09 | Too Low |
| 5/09/2022 | E11 | 0.12 | 0.13 | Too Low | Too Low |
| 5/09/2022 | E12 | 1.07 | Too Low | Too Low | Too Low |
| 5/09/2022 | E13 | 0.51 | Too Low | Too Low | Too Low |
| 5/09/2022 | E21 | 0.09 | Too Low | Too Low | Too Low |
| 5/09/2022 | E22 | 0.08 | Too Low | Too Low | Too Low |
| 5/09/2022 | E23 | 0.48 | 0.051 | Too Low | Too Low |
| 5/09/2022 | L2 | 0.59 | Too Low | Too Low | Too Low |
| 5/09/2022 | E14 | 0.57 | Too Low | Too Low | Too Low |
| 5/09/2022 | E15 | 0.27 | Too Low | Too Low | Too Low |
| 17/03/2023 | L10 | 0.83 | Too Low | Too Low | Too Low |
| 17/03/2023 | E28 | 0.49 | 0.87 | 0.07 | Too Low |
| 17/03/2023 | E29 | 0.18 | 3.78 | Too Low | Too Low |
| 17/03/2023 | L24 | 0.63 | 0.07 | 0.06 | Too Low |
| 17/03/2023 | L11 | 3.42 | 0.06 | 0.13 | 0.41 |
| 17/03/2023 | E30 | 4.86 | 0.12 | 0.08 | 0.28 |
| 17/03/2023 | L25 | 1.56 | Too Low | Too Low | 0.30 |
| 17/03/2023 | L26 | 1.05 | Too Low | Too Low | 0.06 |
| 20/03/2023 | L3 | 0.55 | Too Low | Too Low | Too Low |
| 20/03/2023 | L15 | 0.17 | Too Low | Too Low | Too Low |
| 20/03/2023 | L16 | 0.43 | Too Low | Too Low | Too Low |
| 20/03/2023 | L4 | 0.39 | 0.10 | Too Low | 0.06 |
| 20/03/2023 | L17 | 0.43 | Too Low | Too Low | Too Low |
| 20/03/2023 | L5 | 0.58 | Too Low | Too Low | Too Low |
| 20/03/2023 | L18 | 0.54 | Too Low | Too Low | Too Low |
| 20/03/2023 | L6 | 0.44 | Too Low | Too Low | Too Low |
| 20/03/2023 | E24 | 0.21 | Too Low | Too Low | Too Low |
| 23/03/2023 | E27 | 0.52 | Too Low | Too Low | 0.05 |
| 23/03/2023 | L19 | 1.38 | 1.48 | 0.07 | 0.71 |
| 23/03/2023 | L20 | 0.50 | Too Low | 0.06 | Too Low |
| 23/03/2023 | L21 | 1.26 | 0.09 | Too Low | Too Low |
| 23/03/2023 | L7 | 0.45 | Too Low | 0.05 | Too Low |
| 23/03/2023 | L8 | 0.11 | Too Low | Too Low | Too Low |
| 23/03/2023 | L22 | 0.63 | Too Low | Too Low | 0.06 |
| 23/03/2023 | L9 | 5.28 | Too Low | 0.09 | 0.58 |
| 23/03/2023 | L23 | 1.87 | 0.06 | 0.07 | 0.17 |
| 13/04/2023 | E25 | 1.06 | Too Low | Too Low | 0.06 |
| 13/04/2023 | E26 | Too Low | Too Low | Too Low | Too Low |
| 13/04/2023 | L27 | 0.40 | 0.05 | Too Low | Too Low |
| 13/04/2023 | L28 | 0.26 | Too Low | Too Low | Too Low |
| 13/04/2023 | L12 | 1.23 | 0.61 | 0.06 | Too Low |
| 13/04/2023 | L13 | 0.74 | 0.10 | 0.06 | 0.06 |
| 13/04/2023 | L14 | 0.17 | Too Low | Too Low | Too Low |

**Supplementary Table 2:**

| **LUAD** | **KRAS mutation status** | **UR** | **SUP Total events (WT + MT)** | **P1 Total events (WT + MT)** | **P2 Total events (WT + MT)** | **P3 Total events (WT + MT)** |
| --- | --- | --- | --- | --- | --- | --- |
| **Early-stage** | **Wildtype** | **range** | 116-3682 | 0-2540 | 0-12 | 0-269 |
|  |  | **median** | 344 | 11 | 2 | 5 |
|  |  | **mean** | 596.4 | 186.5 | 2.9 | 26.0 |
|  |  | **SD** | 885.5 | 651.5 | 3.1 | 67.9 |
|  | **Mutant** | **range** | 36-1115 | 3-3055 | 0-193 | 0-40 |
|  |  | **median** | 355 | 25 | 2 | 8 |
|  |  | **mean** | 417.9 | 275.3 | 16.9 | 11.5 |
|  |  | **SD** | 292.8 | 789.9 | 49.1 | 11.0 |
| **Late-stage** | **Wildtype** | **range** | 2-1552 | 0-1061 | 0-54 | 0-294 |
|  |  | **median** | 455 | 13 | 3.5 | 15.5 |
|  |  | **mean** | 591.9 | 88.4 | 13.6 | 46.5 |
|  |  | **SD** | 450.7 | 280.3 | 18.2 | 81.5 |
|  | **Mutant** | **range** | 90-5376 | 0-60 | 1-68 | 0-648 |
|  |  | **median** | 547.5 | 12.5 | 8 | 20 |
|  |  | **mean** | 1246.4 | 21.2 | 20.4 | 69.7 |
|  |  | **SD** | 1594.3 | 19.8 | 24.0 | 168.2 |

**REFERENCES:**

1 Vol. Bulletin 6628 (Bio-Rad Laboratories ).
